# Design of Mesoporous Silica Nanoparticles for the Treatment of Amyotrophic Lateral Sclerosis (ALS) with a Therapeutic Cocktail Based on Leptin and Pioglitazone

**DOI:** 10.1021/acsbiomaterials.2c00865

**Published:** 2022-10-14

**Authors:** Diana Díaz-García, Águeda Ferrer-Donato, José M. Méndez-Arriaga, Marta Cabrera-Pinto, Miguel Díaz-Sánchez, Sanjiv Prashar, Carmen M. Fernandez-Martos, Santiago Gómez-Ruiz

**Affiliations:** † COMET-NANO Group, Departamento de Biología y Geología, Física y Química Inorgánica, E.S.C.E.T., Universidad Rey Juan Carlos, Calle Tulipán s/n, E-28933 Móstoles, Madrid, Spain; ‡ Neurometabolism Group, Research Unit of the National Hospital of Paraplegics (UDI-HNP), Finca La Peraleda s/n, 45071 Toledo, Spain; § Wicking Dementia Research and Education Centre, College of Health and Medicine, University of Tasmania, Hobart, Tasmania 7005, Australia

**Keywords:** amyotrophic lateral sclerosis, mesoporous silica nanoparticles, drug delivery, leptin, pioglitazone

## Abstract

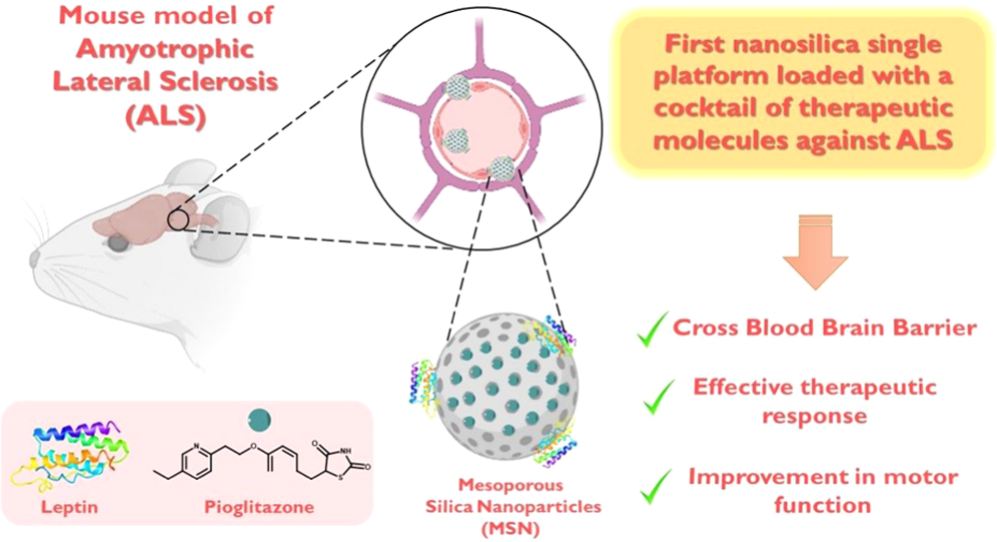

Amyotrophic lateral sclerosis (ALS) is a devasting neurodegenerative disease with no cure to date. Therapeutic agents used to treat ALS are very limited, although combined therapies may offer a more effective treatment strategy. Herein, we have studied the potential of nanomedicine to prepare a single platform based on mesoporous silica nanoparticles (MSNs) for the treatment of an ALS animal model with a cocktail of agents such as leptin (neuroprotective) and pioglitazone (anti-inflammatory), which have already demonstrated promising therapeutic ability in other neurodegenerative diseases. Our goal is to study the potential of functionalized mesoporous materials as therapeutic agents against ALS using MSNs as nanocarriers for the proposed drug cocktail leptin/pioglitazone (**MSN-LEP-PIO**). The nanostructured materials have been characterized by different techniques, which confirmed the incorporation of both agents in the nanosystem. Subsequently, the effect, *in vivo*, of the proposed drug cocktail, **MSN-LEP-PIO**, was used in the murine model of TDP-43 proteinopathy (TDP-43^A315T^ mice). Body weight loss was studied, and using the rotarod test, motor performance was assessed, observing a continuous reduction in body weight and motor coordination in TDP-43^A315T^ mice and wild-type (WT) mice. Nevertheless, the disease progression was slower and showed significant improvements in motor performance, indicating that TDP-43^A315T^ mice treated with **MSN-LEP-PIO** seem to have less energy demand in the late stage of the symptoms of ALS. Collectively, these results seem to indicate the efficiency of the systems *in vivo* and the usefulness of their use in neurodegenerative models, including ALS.

## 1 Introduction

During the last few decades, the interest in developing innovative nanosystems with improved properties has increased undoubtedly due to the enormous potential of these materials in overcoming many of the challenges associated with human progress. Nanomaterials have thus shown great impact in diverse fields, being extensively used in biomedicine, catalysis, photocatalysis, and energy and environmental protection.^1,2^

The implementation of nanomaterials in medicine, also known as nanomedicine, is helping to find solutions to several important issues arising from conventional therapies or diagnostic tests and generating new effective, versatile, reliable, and cost-effective nanosystems, which are capable of overcoming most of the biophysical, biomedical, and biochemical obstacles of the human body and those due to different diseases,^3^ acting in most cases as drug-delivery platforms that safely transport therapeutic or imaging agents to their biological targets.^4^

Even though the use of nanomedicine has led to significant breakthroughs for the treatment or diagnoses of several diseases such as cancer,^5,6^ cardiovascular dysfunctions,^7,8^ or different kind of infections or immune processes,^9,10^ the implementation of nanocarriers in neurodegenerative diseases has not been fully exploited, although is under expansion and continuous development.^11−15^ This group of illnesses affects the nervous system directly, and presently, there is no effective clinical treatment to cure or stop the pathology progression. Therapeutic innovation through nanocarriers or other nanosystems is an urgent priority in this biomedical area.

In this context, ALS is a damaging, irreversible neurodegenerative disease that usually advances with the loss of upper and lower motor neurons of, principally, the brainstem, spinal cord, and cerebral cortex.^16^ In general, it has been estimated that the prevalence of this devastating neurological disease is 5 per 100 000 in the United States. In addition, considering the general population of Europe, it is estimated that ca. two to three people per 100 000 may be affected. To settle this fatal disease in the right context, it is important to note that more than 60% of patients die between 3 and 5 years after the diagnosis of ALS. So far, ALS has no cure as the current research in the field has been unable to effectively stop or decrease its neurodegenerative progression, in addition, there are very limited therapies helping ALS patients to improve the quality and length of their lives.^17^ Therefore, the scientific community is in urgent search of alternative improved therapeutic approaches to treat ALS.

In this context, although some significant therapeutic advances for ALS treatment have been observed, to date, there are only two FDA-approved drugs, namely, Riluzole and Edaravone. However, none of them is able to reverse the neurodegenerative progression of ALS. More than two decades after Riluzole was first approved for ALS in 1995, a more efficacious treatment is yet to be discovered. However, there is hope in the field of ALS that neuroprotective and anti-inflammatory agents counteracting excitotoxicity, oxidative stress, inflammatory damage, or other pathogenic mechanisms might ameliorate the clinical symptoms of ALS. Indeed, there has been a call in the ALS research community to examine the use of combination therapy in ALS to tackle multiple pathological mechanisms.

Thus, previous studies have determined how pioglitazone, a drug of the thiazolidinedione (TZD) family, has demonstrated in several murine models to lead to anti-inflammatory and neuroprotective effects in ALS.^18,19^ In addition, leptin, which is a polypeptide hormone primarily secreted by adipocytes and regulates energy balance and food intake in the brain,^20,21^ has been demonstrated to act as a neuroprotective species reducing the progressive deterioration of neurological conditions. Several trials point toward an advantageous effect of leptin on Alzheimer’s disease (AD) by enhancing cognitive and learning functions.^22^ Therefore, most of the neurological effects associated with leptin are currently being tested in other neurodegenerative diseases, such as Parkinson’s disease,^23^ to have robust experimental evidence of the potential therapeutic role of leptin. Moreover, it has been observed in some epidemiological and clinical research works that altered leptin levels may be implicated in ALS pathogenesis.^24^ Indeed, recent epidemiological work has associated ALS risk with low altered levels of leptin.^25^ In addition, patients with frontotemporal dementia (FTD) and ALS^26^ have shown altered peripheral levels of leptin, which usually happen in the continuous clinical advances of ALS.^27^

Some recent reports have demonstrated that a therapeutic combination of leptin and pioglitazone as a single-target approach in the treatment of neurodegenerative conditions may be beneficial in neurological disorders therapy.^28,29^ Therefore, although ALS has not been extensively targeted by the use of therapeutic nanomaterials,^30^ the use of nanoplatforms for the delivery of cocktails of drugs, such as a mixture of leptin and pioglitazone, may help to improve the efficiency of this combined therapeutic approach.

In this present work, we have focused on the preparation of mesoporous silica nanoparticles (MSNs) as nanocarriers for the proposed drug cocktail leptin/pioglitazone (**MSN-LEP-PIO**). MSNs have been chosen because they have already been widely employed as carriers for a diversity of therapeutic agents, becoming lead nanomaterial in drug delivery.^31−33^ Nevertheless, in the field of neurodegenerative diseases, there is only one study on the use of silica nanoparticles for ALS treatment,^34^ and the results were not encouraging because the results showed unexpectedly that the unmodified silica nanoparticles were more active against ALS than their analogues containing therapeutic molecules. Therefore, we hypothesized that the capacity of MSN nanocarriers to help in the protection and delivery of both pioglitazone and leptin drugs might be very useful for treating ALS in a simple and functional way, controlling both therapeutic agents in a low toxicity multifunctional platform.^34^

Bearing in mind that the use of silica nanocarriers has not been explored in depth in ALS and other comparable neuronal illnesses, the preliminary results reported here may open up new avenues in the field of drug delivery at neurological level, studying the biocompatibility of the functionalized materials and their potential capacity to cross the blood–brain barrier (BBB).^35^

Thus, to the best of our knowledge, we report one of the first examples of effective treatment of ALS by silica-based drug delivery of nanomaterials to biological targets in the central nervous system.^30,36^ Our results open up promising new possibilities in the fight against the symptoms and fatal progression of this disease.

## 2 Materials and Methods

### 2.1 Synthesis and Characterization of the Materials

The reagents used in the preparation of the materials were 1-ethyl-3-(3-dimethylaminopropyl)carbodiimide hydrochloride (EDAC), hexadecyltrimethylammonium bromide (CTAB), tetraethyl orthosilicate (TEOS), N-hydroxysuccinimide (NHS), (3-aminopropyl)triethoxysilane (AP), leptin from mouse (LEP), and pioglitazone hydrochloride (PIO). All were purchased from Aldrich and used as received with no additional purification.

X-ray diffraction (XRD) patterns were recorded on a Philips Diffractometer model PW3040/00 X’Pert MPD/MRD at 45 kV and 40 mA, using a wavelength Cu Kα (λ = 1.5418). Adsorption–desorption isotherms of nitrogen were measured using a Micromeritics ASAP 2020. The surface areas and the pore size were calculated by the BET and BJH methods, respectively. Thermogravimetry (TG) analyses were performed with a Shimadzu model at a heating rate of 20 °C/min from 30 to 800 °C under nitrogen. Transmission electron microscopy (TEM) images were obtained with a JEOL JEM 1010 at a 100 kV operating voltage, and the micrographs were treated using ImageJ software. Scanning electron microscopy (SEM) was performed using an XL30 ESEM Philips with a high-resolution FEG-SEM Nova Nano SEM230. Diffuse reflectance ultraviolet–visible (DR UV–vis) spectra were obtained using a Varian Cary-500 spectrophotometer equipped with an integrating sphere and poly(tetrafluoroethylene) (PTFE) as a reference. ^13^C cross-polarization (^13^C-CP MAS NMR) spectra (4.40 μs 90° pulse, spinning speed of 6 MHz, pulse delay 2 s) and ^29^Si magic angle spinning nuclear magnetic resonance (^29^Si MAS NMR) spectra (8 μs 90° PDA, spinning speed of 6 MHz, pulse delay 10 s) were recorded on a Varian-Infinity Plus Spectrometer at 400 MHz operating at a 100.52 MHz frequency. An ICP-AES study was carried out on a Varian Vista AX Pro Varian 720-ES (λ_Si_ = 250.69 nm). Circular dichroism (CD) studies were carried out on a JASCO J-815 spectrometer at 37 °C, with a scanning speed of 50 nm/min between 200 and 260 nm. The samples were prepared in PBS buffer at pH 7.4 in 5 mm glass cuvettes.

### 2.2 Synthesis of Mesoporous Silica Nanoparticles (MSNs)

For the preparation of MSNs, slight modifications of the sol–gel method reported by Zhao et al.^37^ were carried out. In brief, 3.5 mL of a 2 M aqueous sodium hydroxide solution was added to a solution of CTAB (1.0 g, 2.74 mmol) in 480 mL of Milli-Q water. Subsequently, the temperature of the reaction mixture was increased to 80 °C, and then, the silica precursor TEOS (5 mL, 22.4 mmol) was added dropwise under vigorous stirring, allowing the mixture to react for 2 h. After this time, the white precipitate was isolated by filtration, washed with abundant Milli-Q water and methanol (2 × 20 mL), and dried for 24 h at 80 °C on a stove. Finally, a calcination process at 550 °C was performed for 24 h with an increasing temperature ramp of 1 °C/min.

### 2.3 Functionalization with (3-Aminopropyl)triethoxysilane (AP)

The incorporation of the AP ligand was carried out following the procedures reported by our research group.^38^ First, 500 mg of MSN was heated at 90 °C under a vacuum overnight in a Schlenk tube. The resulting dehydrated material was then dispersed in 25 mL of dry toluene, and subsequently, 105.7 μL of AP (20% w/w AP/SiO_2_) was added to the solution. The mixture was then heated to 110 °C and stirred at this temperature for 48 h. Finally, the dispersion was centrifuged, and the solid product was washed with toluene and diethyl ether. The resulting white solid, **MSN-AP**, was then dried overnight on a stove at 75 °C. Once the AP-loaded system was synthesized, the concentration of leptin and pioglitazone for the subsequent functionalization reactions was adjusted to the quantities previously tested by our group in other preclinical studies;^28,29^ see Sections 2.4 and 2.5 for further details.

### 2.4 Covalent Incorporation of Leptin (LEP)

**Scheme 1:**
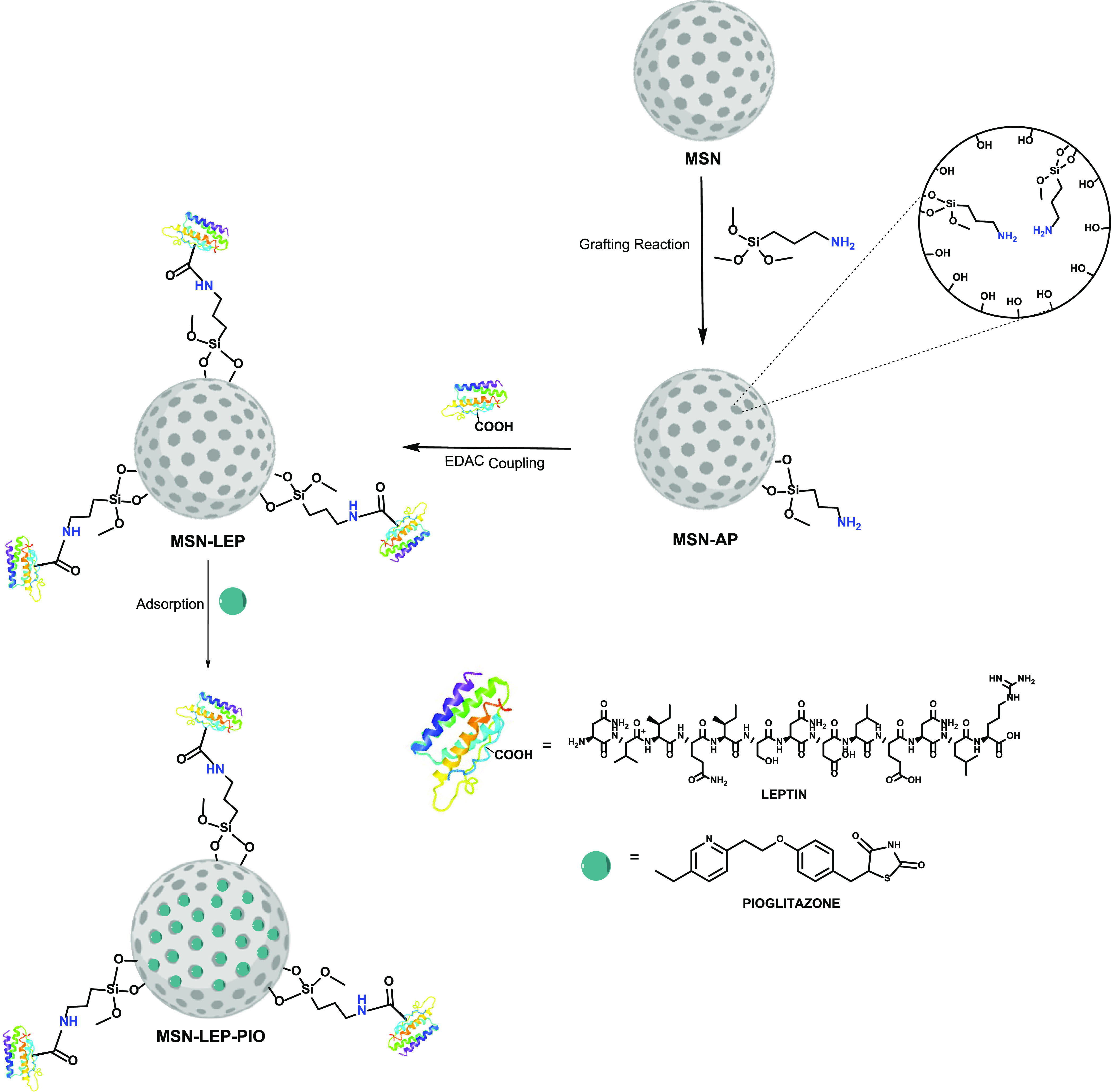
Synthetic Route for the Synthesis of MSN-Functionalized Materials

For the functionalization of **MSN-AP** with leptin (Scheme 1), an EDAC coupling reaction was carried out. A total of 1 mg of leptin from a mouse (1% w/w SiO_2_/LEP) was dissolved in 10 mL of MES buffer 0.1 M. Then, 4 mg of EDAC (0.02 mmol) and 6 mg of NHS (0.05 mmol) were added to the leptin solution and left under vigorous stirring for 30 min. Subsequently, 100 mg of **MSN-AP** was added, and the mixture was stirred for an additional 2 h at room temperature. The final material **MSN-LEP** was centrifuged and washed with ethanol (2 × 20 mL).

### 2.5 Pioglitazone (PIO) Adsorption or Encapsulation

PIO was incorporated into the silica material by a simple adsorption method. Initially, 100 mg of **MSN-LEP** was dispersed in 10 mL of ethanol. After 15 min of vigorous stirring, 10 mg of PIO (0.025 mmol) was added to obtain 10% w/w SiO_2_/PIO in the final material **MSN-LEP-PIO** (Scheme 1). The mixture was stirred at room temperature for 24 h, and subsequently, the suspension was centrifuged, and the isolated solid was dried under a vacuum.

### 2.6 Release Study of Pioglitazone

To analyze the release kinetics of the drug adsorbed in the silica, an incubation study of the materials **MSN-PIO** and **MSN-LEP-PIO** was carried out. The experimental procedure was as follows: a suspension of 3 mg of the studied material in 3 mL of buffer PBS 7.4 was incubated at 37 °C in a Roto-Therm Plus incubator for 24 and 72 h. After that time, the suspension was filtered with a 0.22 μm nylon filter, and the liquid was mixed with acetonitrile (ACN) at a ratio of 50:50 and subsequently analyzed by high-performance liquid chromatography (HPLC Flexar Perkin-Elmer) using a C18 column (COSMOSIL 5C18-MS-II 4.6 mm I.D. × 250 mm with a particle size of 4.4 μm) with a mobile phase composed of ACN:H_2_O (50:50); the flow rate was 0.8 mL/min and the UV–vis detection wavelength was 228 nm. Under these conditions, pioglitazone appears at a peak at 6.2 min.

### 2.7 Preparation of the Samples for *In Vivo* Studies

All of the final materials were redispersed in a PBS buffer with a concentration of 10 mg/mL immediately before the *in vivo* assays, maintaining them at 4 °C before the injection.

### 2.8 Preliminary *In Vivo* Preclinical Study

#### 2.8.1 Experimental Design and Drug Treatments

**Scheme 2:**
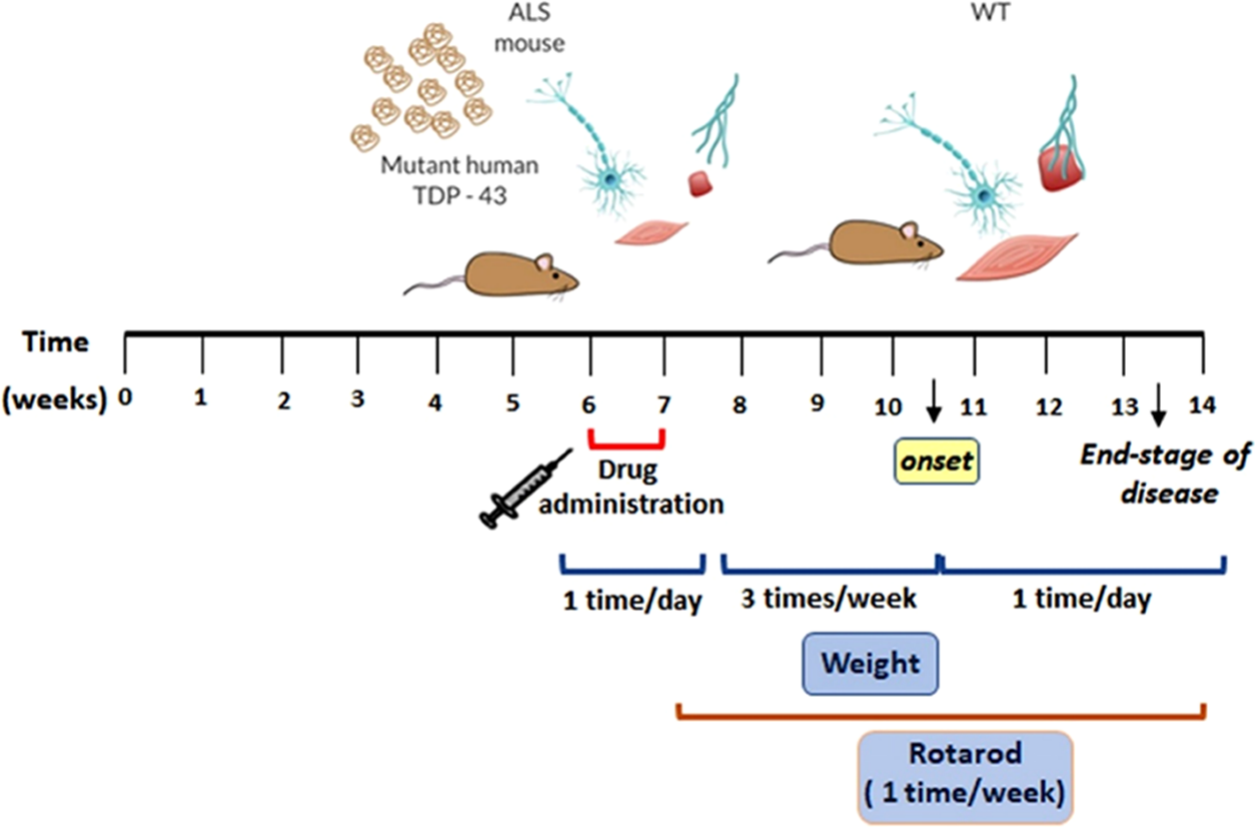
Schedule of the Experiment Design

In this work, cohorts of male TDP-43^A315T^^39^ and the genetic background-matched wild-type (WT) littermate control mice were randomly distributed into two experimental subgroups (*n* = 3–6 TDP-43^A315T^ mice/subgroup and *n* = 3 WT mice/subgroup) and treated with drugs according to the treatment (Scheme 2). Beginning at 42 days of age (asymptomatic phases of disease), animals were treated with an intraperitoneal (IP) injection of 10 mg/mL of **MSN-LEP-PIO** or PBS (pH 7.2) daily for 7 consecutive days. Additionally, two mice were treated IP with **MSN-AP** (*n* = 1 mice/genotype). The ALS-like disease was divided into three stages according to time points: asymptomatic (40–42 days), preonset (60–70 days), and early end-stage of disease (90–95 days), defined as the duration of time between peak body weight until the loss of 20% of peak body weight. Thus, to monitor disease progression, mice were weighed and assessed three times per week until the disease onset stage. After this, mice were then checked daily in the morning until the disease end stage (Scheme 2). We selected the IP administration route because it is commonly used as a noninvasive drug administration technique, which promotes minimal discomfort. IP injection is commonly used in smaller mammals for which intravenous access is challenging.^40^ Mice were closely monitored in terms of their mobility or level of activity immediately after the IP procedure. No adverse effect of MSN on body weight, a biological indicator of general health, was determined (data not shown). During the 7 days of IP drug treatment, no differences in weight gain between groups (**MSN-LEP-PIO** group vs PBS group) were displayed (Table 1). The maintenance and use of mice and all experimental procedures were approved by the Animal Ethics Committee of the National Hospital for Paraplegics (HNP) (Approval No. 26/OH 2018), in accordance with the Spanish Guidelines for the Care and Use of Animals for Scientific Purposes. Drug administrations were conducted by personnel blinded to the animal genotype.

**Table 1:** No Difference in Body Weight Gain was Observed in TDP-43^A315T^ Mice and WT Littermate Controls Treated with MSN-LEP-PIO vs PBS^a^

	1 day	2 days	3 days	4 days	5 days	6 days	7 days
WT VH (*n* = 3)	20.09 ± 0.20	20.63 ± 0.35	21.63 ± 0.32	21.90 ± 0.43	21.87 ± 0.51	22.13 ± 0.47	21.65 ± 0.26
WT L+P (*n* = 3)	20.10 ± 0.88	19.60 ± 0.70	20.10 ± 0.79	21.13 ± 0.90	20.83 ± 1.30	21.20 ± 0.95	20.42 ± 0.69
TDP-43^A315T^ VH (*n* = 3)	18.93 ± 0.49	18.80 ± 0.78	18.87 ± 0.83	19.03 ± 0.77	19.40 ± 0.62	19.50 ± 0.69	19.22 ± 0.63
TDP-43^A315T^ L+P (*n* = 6)	20.17 ± 1.07	19.43 ± 0.97	20.17 ± 0.96	20.63 ± 0.80	20.33 ± 1.12	20.67 ± 0.84	20.46 ± 0.63
WT VH (*n* = 3)	20.09 ± 0.20	20.63 ± 0.35	21.63 ± 0.32	21.90 ± 0.43	21.87 ± 0.51	22.13 ± 0.47	21.65 ± 0.26

a Body weight was monitored daily in WT and TDP-43^A315T^ mice during the 7 day treatment period, and no difference in body weight gain between treatments and groups was observed. Values are expressed as mean ± SEM.

#### 2.8.2 Functional Evaluation

Motor performance, coordination, and strength were evaluated using the rotarod test.^41,42^ Animals were habituated to the test room and human handling prior to being placed on a rotarod apparatus (Model 7650, Ugo Basile). Then, mice were trained three times a week to promote the learning of the task and then tested weekly^43^ using an accelerated protocol,^44^ beginning at 42 days of age (∼6 weeks of age) until the day of euthanasia. Three tests were performed for each mouse with a minimal interval of 20 min over a maximum time of 300 s. The average of the longest two performances was taken as the final result for analysis, which was conducted by personnel blinded to the animal genotype.

#### 2.8.3 Tissue Preparation and Silicon Determination

Animals were terminally anesthetized with sodium pentobarbitone (140 mg/kg) and transcardially perfused with 0.01 M phosphate-buffered saline (PBS; pH 7.4) Motor cortex and lumbar spinal cord (L4–L6) from each animal were processed to extract proteins for silicon determination. Samples were immediately frozen on dry ice and stored at −80 °C for later analysis.

Protein samples were treated using the following procedure to analyze the presence of silicon in different tissue samples. For each sample, 2 mL of concentrated HNO_3_ was added, and the solution was heated to 65 °C and stirred for 24 h. In a second step, 1 mL of concentrated HF and 1 mL of concentrated HCl were added at room temperature for an additional 24 h.

#### 2.8.4 Biological Statistical Analysis

All data are presented as mean ± standard error of the mean SEM, and differences are considered significant at *p* < 0.05 (CI 95%). Differences between groups were evaluated using two-way ANOVA followed by Dunnett’s post hoc test to compare all groups with control WT onset mice, and Tukey’s post hoc test was used for multiple comparisons between all groups. Statistical analysis was performed using GraphPad Prism software (version 6.0).

## 3 Results and Discussion

### 3.1 Preparation and Structural and Morphological Characterization of the Materials

The material design is based on a drug cocktail of leptin^45^ and pioglitazone functionalized in MSNs with the purpose of targeting ALS-affected animals. In this context, first, the ligand (3-aminopropyl)triethoxysilane was incorporated into the MSNs to have pendant primary amines, which can react with the carboxylic groups of the leptin via EDAC coupling. In addition, the nonreacted amino ligands will also be useful in the last step of the adsorption of pioglitazone as they may favor intermolecular interactions with the heteroatoms of the thiazolidine-2,4-dione fragment of PIO. The extensive characterization of the systems by different methods, such as powder XRD, BET, TG, and solid-state ^13^C and ^29^Si NMR spectroscopy and FTIR spectroscopy, confirmed the incorporation of both therapeutic molecules LEP and PIO in the silica nanomaterials.

**Figure 1:**
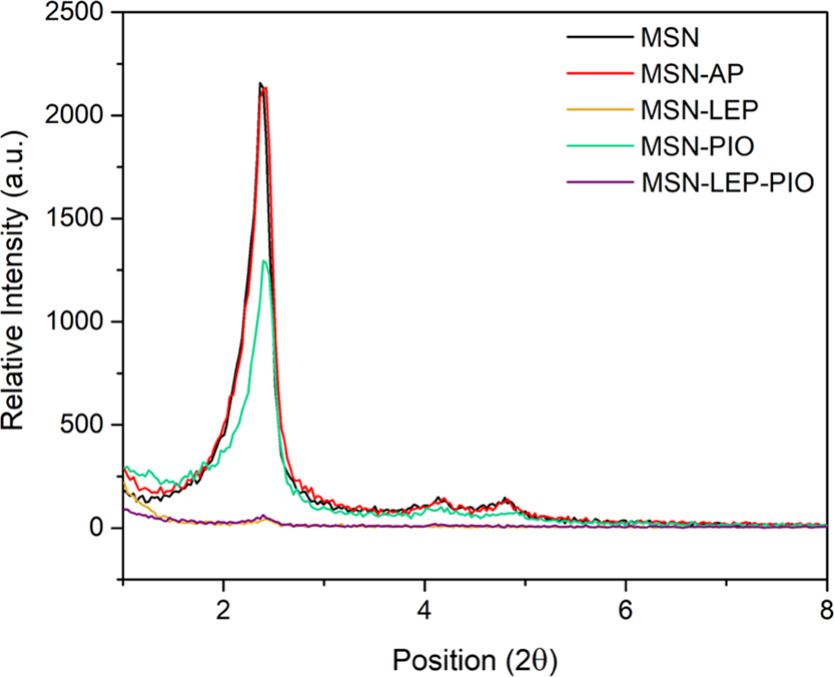
XRD diffraction patterns of MSN, MSN-AP, MSN-LEP, MSN-PIO, and MSN-LEP-PIO.

All of the MSN-based nanomaterials were characterized by powder X-ray diffraction measurements (Figure 1). The diffraction patterns show the peaks associated with mesoporous silica with a hexagonal pore distribution that corresponds to the MSN system. The diffraction peak associated with the Miller plane (100) appears at ca. 2.36°, and two small peaks at ca. 4.13 and 4.80°, associated with the planes (110) and (200), respectively, were also observed. It is interesting to note that the functionalization with 20% of AP (**MSN-AP**) does not produce any significant change in the diffractogram compared to the starting material. However, the incorporation of leptin (**MSN-LEP** and **MSN-LEP-PIO**) provokes an enormous decrease in the relative intensity of the signals of the diffraction pattern. This is due to the blocking of the silica pores caused by leptin being larger (ca. 8.8 × 8.8 × 4.8 nm^3^) than the pore diameter of the MSN (less than 3 nm). The incorporation of leptin prevents the intense and ordered diffraction of the pore walls, consequently reducing the intensity of the peak.

**Figure 2:**
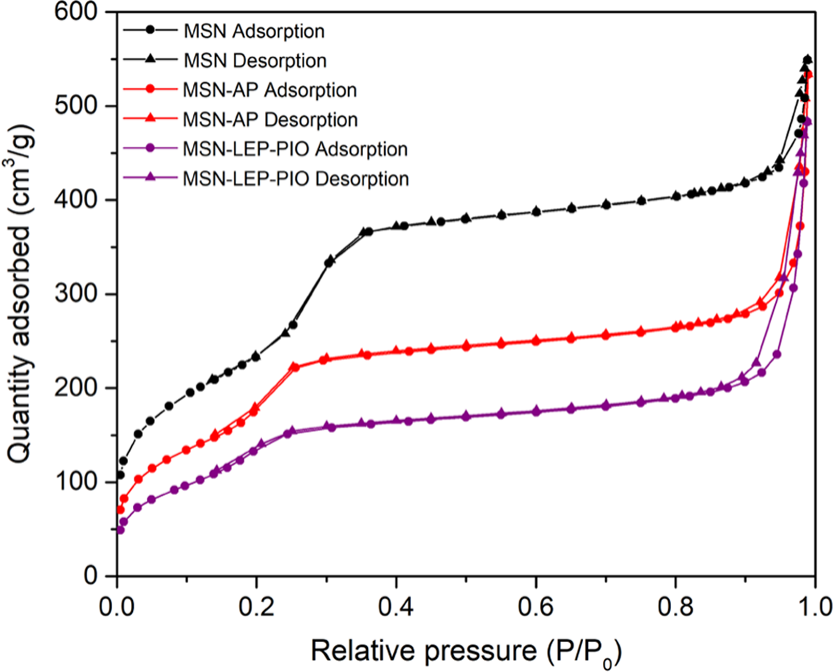
Nitrogen adsorption–desorption isotherms of MSN, MSN-AP, and MSN-LEP-PIO.

The N_2_ adsorption study (Figure 2) gives isotherms between type IV and type VI for the unloaded MSN,^46^ highlighting the mesoporous nature of the material (surface area of 853 m^2^/g, a pore diameter of 3.42 nm, and a pore volume of 0.73 cm^3^/g). After the incorporation of AP, a clear decrease in the surface area, pore volume, and pore diameter of the material confirms that the incorporation of the aminopropyl ligand takes place both on the external surface and inside the pores (see Scheme 1). Comparing both AP-modified materials (Table 2), the values of surface area, pore volume, and pore diameter are in similar ranges, which means that the differences in the functionalization rate of AP do not have a dramatic impact on the final porosity of the material.

**Table 2:** Textural Parameters Obtained by TG and BET Studies

Material	%AP^a^	*S*_BET_^b^ (m^2^/g)	*D*_p_^b^ (nm)	*V*_p_^b^ (cm^3^/g)
MSN		853	3.42	0.73
MSN-AP	8.35	651	3.17	0.52
MSN-LEP-PIO	8.35	512		0.47

a Determined by thermogravimetry.

b Determined by BET studies.

Interestingly, the final therapeutic material **MSN-LEP-PIO** containing both agents, leptin and pioglitazone, shows a much lower surface area than that observed for MSN and also lower than those recorded for the AP-modified systems, indicating the successful incorporation of both agents. Thus, **MSN-LEP-PIO** has a surface area of 512 m^2^/g and a pore volume of 0.47 cm^3^/g, while the pore diameter is lower than 2 nm due to the blocking of the pores by leptin.

**Figure 3:**
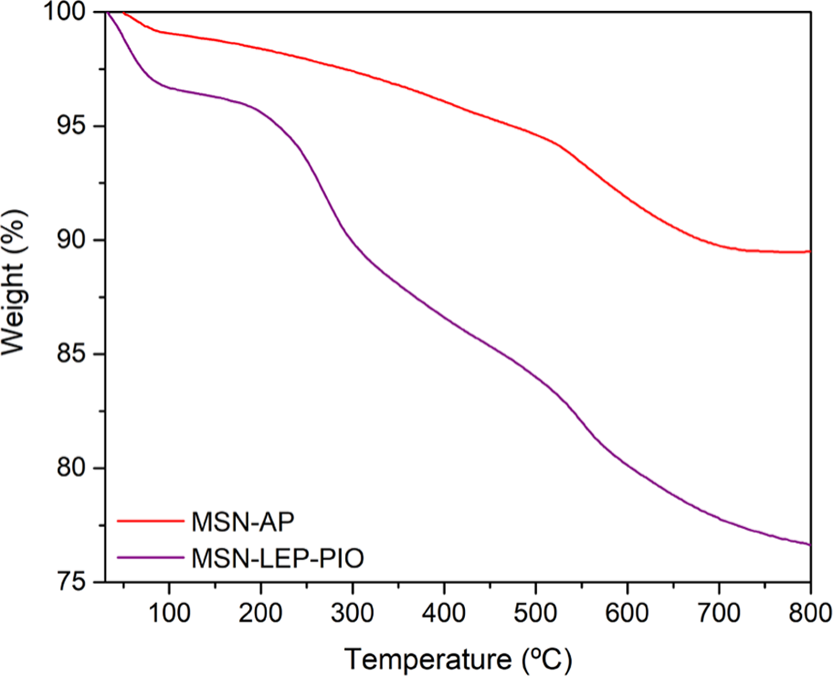
Comparative between MSN-AP and MSN-LEP-PIO thermogravimetric curves.

The thermogravimetric curve between 50 and 800 °C of **MSN-AP** shows a weight loss corresponding to an 8.35% of AP functionalization in the silica support. The TG curve of the final material **MSN-LEP-PIO** exhibits three main stages of weight loss, which appear to correspond with the different incorporated fragments, namely, AP, leptin, and pioglitazone, which has previously been shown to decompose mainly in three different steps^47^ (Figure 3). The leptin degradation overlaps with the pioglitazone degradation in the curves, and this precludes an accurate analysis of the real degree of functionalization of both molecules in the materials.

The MSN-based nanosystems were subsequently characterized by electronic microscopy, observing quasi-spherical particles of a narrow size distribution of 94 ± 15 nm.

**Figure 4:**
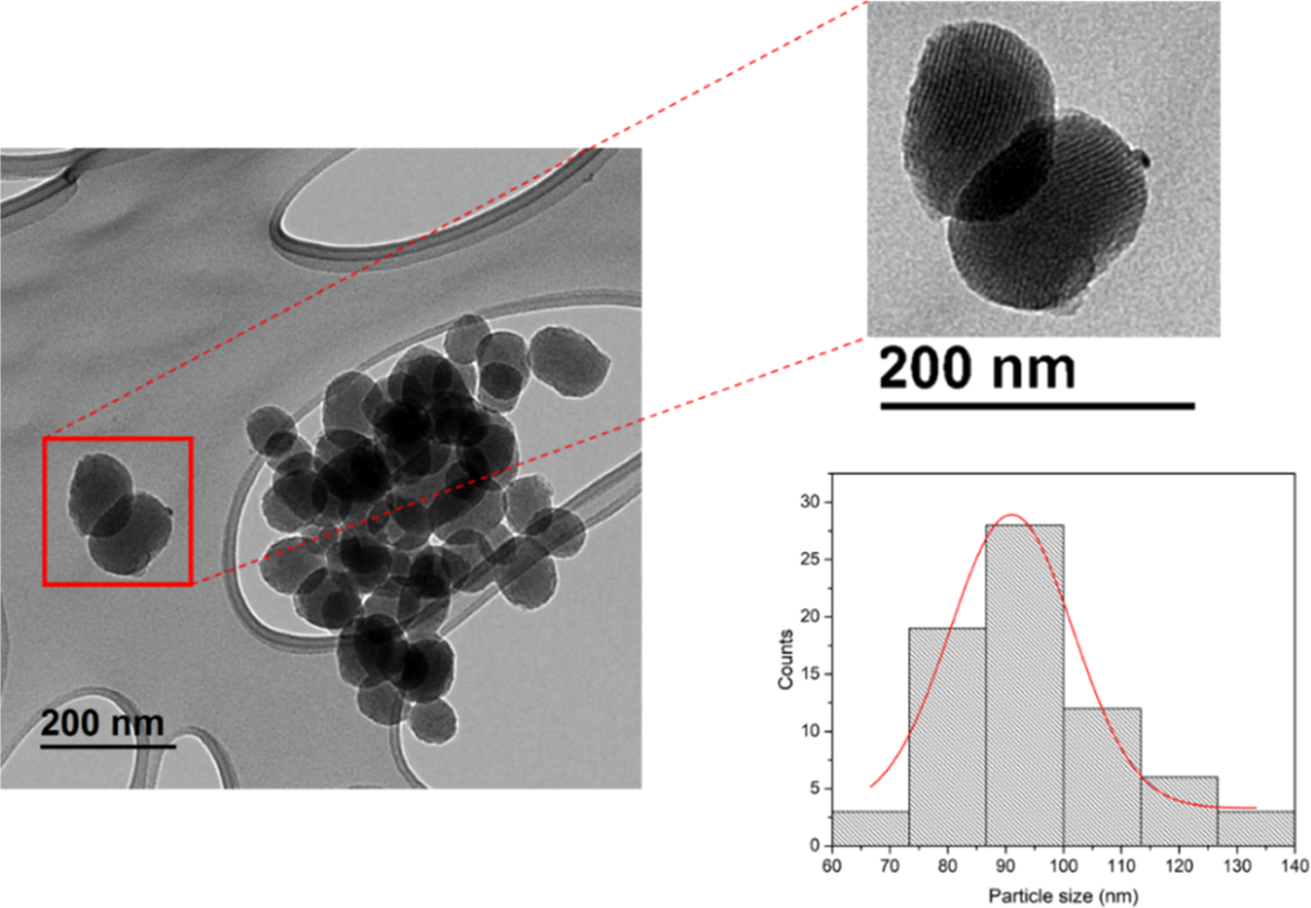
TEM images and particle distribution of MSN nanoparticles.

**Figure 5:**
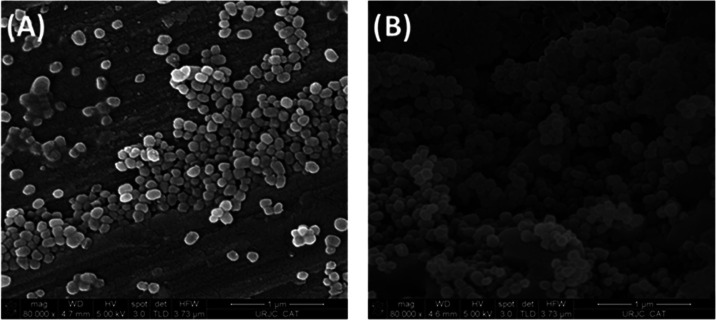
SEM images of (A) MSN starting nanoparticles and (B) MSN-LEP.

The internal silica porous channels can easily be observed in the TEM images (Figure 4), which show an ordered distribution. SEM images also show the homogeneity of the particles (Figures 5a and S1) and confirm that the functionalization does not change the morphology of the nanomaterial (Figures 5b and S2).

**Figure 6:**
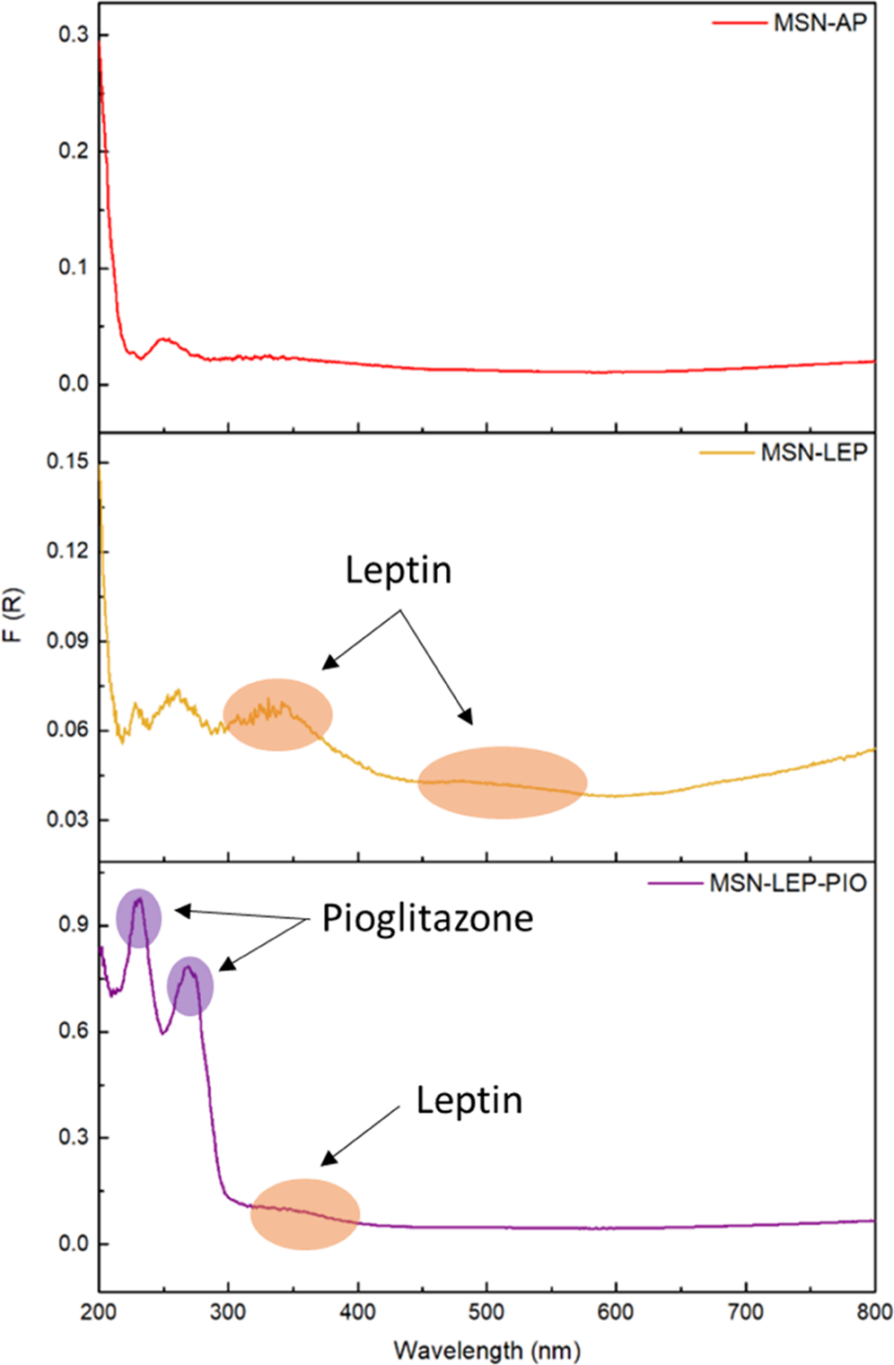
UV spectra of MSN-AP, MSN-LEP, and MSN-LEP-PIO.

Solid-state diffuse reflectance spectroscopic studies of the studied materials were carried out to identify the absorption bands of the supported agents in the nanoparticles (Figure 6). Significant peaks were observed in the 200–400 range. For the material **MSN-LEP** (in comparison with **MSN-AP**), new signals appear at ca. 330 and ca. 500 nm and were attributed to the incorporation of leptin. The final material **MSN-LEP-PIO** shows two intense peaks at 228 and 269 nm assigned to the pioglitazone present in the material and a decrease in the relative intensity of the rest of the signals. The incorporation of LEP and PIO can also be seen in the infrared spectra (Figure S3), where certain bands of the spectrum changed when compared with the starting silica. Between 3000 and 2750 cm^–1^, new peaks appear, corresponding to the bonds C–H and N–H present in both molecules with higher intensity for the **MSN-LEP-PIO** material. The same is observed at 1500–1300 cm^–1^ and around 700 cm^–1^.

**Figure 7:**
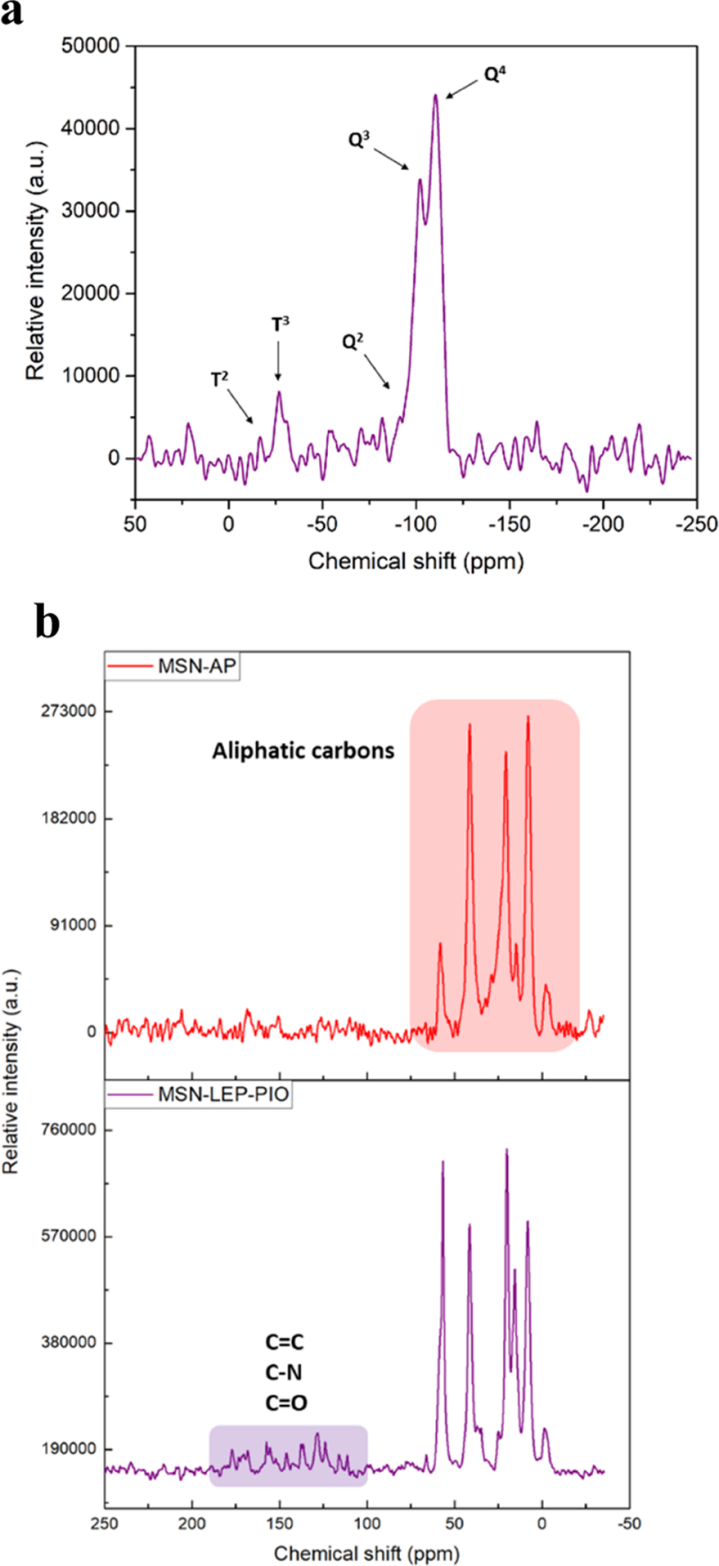
^29^Si NMR MAS spectra of MSN-LEP-PIO (a) and ^13^C NMR MAS spectra of MSN-AP and MSN-LEP-PIO (b).

Solid-state ^29^Si NMR and ^13^C NMR spectra were also studied to identify the successive incorporations of organic molecules into the system after each reaction (Figure 7). The ^29^Si NMR spectrum of **MSN-LEP-PIO** (Figure 7a) shows the typical Q^4^ and Q^3^ intense peaks of the silicon atoms of the silica (corresponding to SiO_4_ and SiO_3_R, respectively) at ca. −110 ppm and the low-intensity peaks associated with the Q^2^ (SiO_2_R_2_) and Q^1^ (SiOR_3_) peaks (barely visible), typical of a silica material. In addition, the ^29^Si NMR spectrum shows the T^2^ (RSi(OSi)_2_(OR′)) and T^3^ (RSi(OSi)(OR′)_2_) peaks at ca. −25 ppm associated with the incorporation of the AP and the leptin fragment because these T-signals correspond to the condensation of organic species on the silica surface. Interestingly, the ^13^C NMR spectra of the different materials also confirm the incorporation of both leptin and pioglitazone in the material (Figure 7b). The spectrum shows the appearance of several broad resonances between 0 and 60 ppm, which correspond to the aliphatic carbon atoms of the AP ligand. Specifically, the first signal, located at 0 ppm, is assigned to the methoxy groups of the corresponding SiOSi(OMe) systems. In addition, the peaks between 4 and 20 ppm are due to the CH_2_–methylene groups of AP. Furthermore, the resonances between 25 and 60 ppm correspond with the carbon atoms of the CH_2_ group adjacent to amino groups. In addition, the spectrum of the final material **MSN-LEP-PIO** shows additional signals between 100 and 200 ppm with less intensity that correspond to the carbon atom of phenyl groups and the aromatic C–N, C=O, and C–S carbon atoms. The assignation of the peaks is not easy for each of the carbon atoms of the therapeutic molecules. However, the appearance of this high number of signals of low intensity between ca. 100 and 200 ppm and the change of intensity of the peaks between 25 and 60 ppm confirm the incorporation of both leptin and pioglitazone.

**Figure 8:**
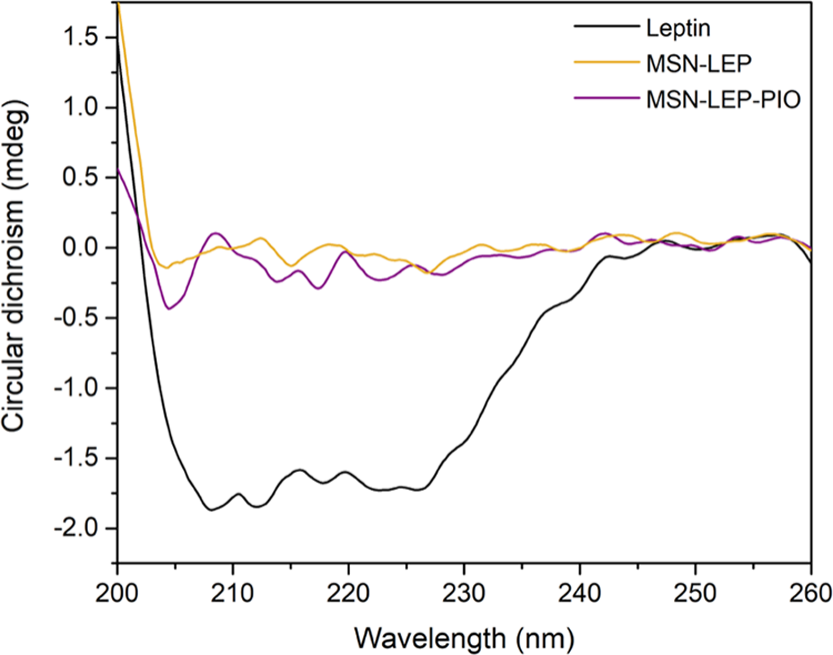
CD spectra evolution of leptin, MSN-LEP, and MSN-LEP-PIO suspensions (0.01 mg/mL in PBS buffer).

To study the conformation of the leptin protein after its incorporation into the silica material, CD spectroscopy was carried out. In Figure 8, it can be observed that **MSN-LEP** and **MSN-LEP-PIO** did not show a significant absorption compared with the leptin solution, which clearly indicates an intense interaction between leptin and MSN after the EDAC coupling reaction and covalent binding.

**Figure 9:**
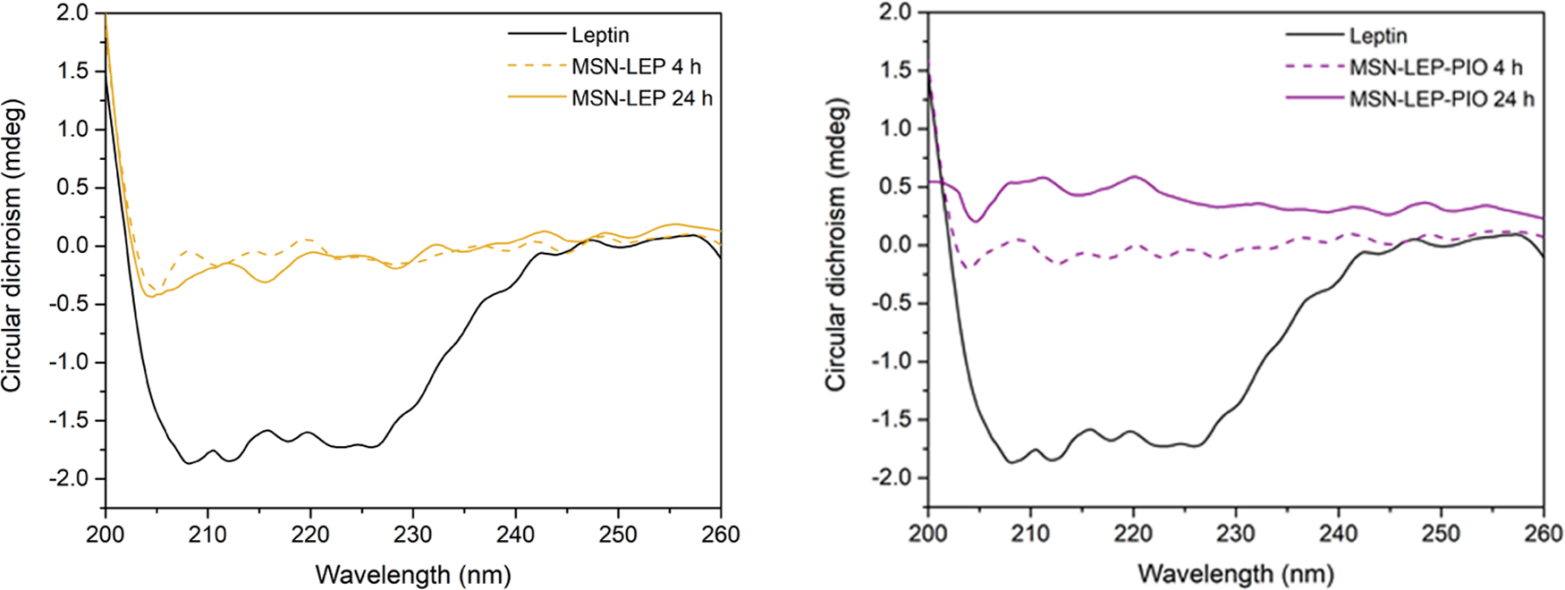
Left: CD spectra comparison between pure leptin (0.01 mg/mL) and MSN-LEP release after 4 and 24 h of incubation. Right: CD spectra comparison between pure leptin (0.01 mg/mL) and MSN-LEP-PIO release after 4 and 24 h of incubation.

To check the strength of this covalent union, release studies were performed with **MSN-LEP** and **MSN-LEP-PIO** materials. Thus, after 4 and 24 h of incubation of **MSN-LEP** and **MSN-LEP-PIO** materials at 37 °C and 30 rpm in PBS buffer, UV spectra were recorded looking for the leptin signal at the maximum absorbance, and no significant bands were noticed. CD spectra were also in line with UV results (Figure 9), with no α-helix structure for release at 4 and 24 h for both materials. These results support the fact that leptin is not easily released in a physiological medium, suggesting the hypothesis of the nonclassical behavior of our materials, which may therapeutically act as a whole entity at the target, not releasing the drug during transport.

The release study of the materials loaded with pioglitazone (**MSN-PIO** and **MSN-LEP-PIO**) shows a low drug release after incubation times under physiological conditions (Table 3). Increasing the incubation time does not show an increase in the amount of drugs released. In addition, it can be observed that the material also functionalized with leptin, leading to a similar release of pioglitazone, indicating good stability and reproducibility of pioglitazone release in the studied materials.

**Table 3:** Release Analysis of Pioglitazone by HPLC

	Release PIO (ppm)	
Material	24 h	72 h
MSN-PIO	1.39	1.12
MSN-LEP-PIO	1.19	1.18

### 3.2 Evaluation of Drug Treatments on Disease Progression in TDP-43^A315T^ Mice

**Figure 10:**
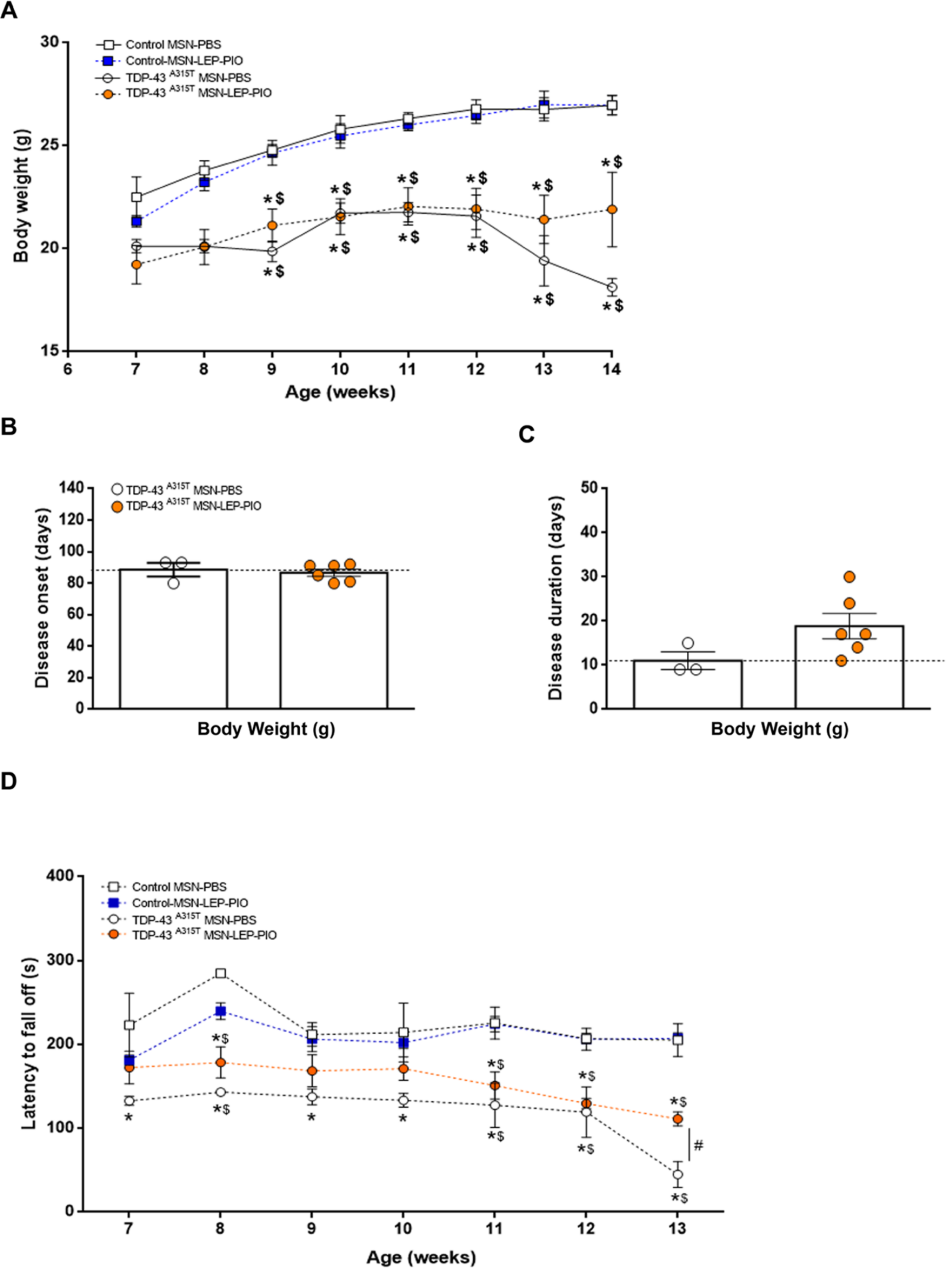
MSN-LEP-PIO treatment beginning at the asymptomatic state of disease significantly enhances motor performance in TDP-43^A315T^ mice. (A) Time monitoring of body weight was carried out in WT controls and TDP-43^A315T^ mice IP treated with MSN-LEP-PIO or PBS. Starting weight in week 7. No significant differences were observed between MSN-LEP-PIO- or PBS-treated TDP-43^A315T^ mice. (B) Average disease onset and disease duration (C) was determined in WT controls and TDP-43^A315T^ mice IP treated with MSN-LEP-PIO or PBS using body weight as a physiological parameter. The average disease duration of the animal was calculated as the time between the onset of the disease (defined as the last day of individual peak body weight before a gradual loss occurs) and the day of death. Comparatively, the disease duration was higher in TDP-43^A315T^ mice in response to MSN-LEP-PIO treatment. (D) Behavioral assessment of the motor function was performed in WT controls and TDP-43^A315T^ mice IP treated with MSN-LEP-PIO or PBS over time. Significant differences between MSN-LEP-PIO- and PBS-treated mice were seen. Values are expressed as mean ± SEM. A comparison between groups was performed by two-way ANOVA, where *p < 0.05 vs PBS-treated WT control mice; ^$^*p* < 0.05 vs MSN-LEP-PIO-treated WT control mice; and ^#^*p* < 0.05 vs MSN-LEP-PIO-treated TDP-43^A315T^ mice. Corresponding graphs as per (A), i.e., control–PBS (*n* = 3, white square and solid line), control–MSN-LEP-PIO (*n* = 3, blue square and dashed line), TDP-43^A315T^–PBS (*n* = 3, white circles and solid line), and TDP-43^A315T^–MSN-LEP-PIO (*n* = 6, orange circles and dashed line).

Monitoring of motor performance and body weight loss was carried out until the day of euthanasia to gain insights into the therapeutic nanosystems of the disease advances. This study was carried out both in TDP-43^A315T^ mice and WT controls IP-treated with **MSN-LEP-PIO** or PBS, beginning at the asymptomatic phases of the disease (Figure 10). Considering that leptin is a fundamental agent for regulating energy balance and body weight,^21,48^ and that previous studies published by us and others have shown that TDP-43^A315T^ mice lose weight when ALS disease advances,^49−53^ we evaluated the capacity of **MSN-LEP-PIO** nanomaterial to alter weight changes during the clinical course of the disease. A two-way ANOVA revealed a significant repercussion of genotype and treatments (*p* < 0.0001, respectively, Figure 10A), pointing to a sustained decline over time in TDP-43^A315T^ mice body weight compared to WT controls in responses to **MSN-LEP-PIO** or PBS. Even though some trend was found, **MSN-LEP-PIO** treatment had no statistically significant effect on weight loss in TDP-43^A315T^ mice (Figure 10A). Using body weight gain, we calculated the disease onset (defined as the last day of individual peak body weight before a gradual loss occurs), and our results indicated that TDP-43^A315T^**MSN-LEP-PIO**-treated mice develop symptoms similar to the PBS-treated TDP-43^A315T^ mice (Figure 10B). Indeed, an average onset of 88 ± 4 days of age was determined in TDP-43^A315T^ mice treated with PBS, whereas **MSN-LEP-PIO**-treated TDP-43^A315T^ mice presented a phenotype at 86 ± 2 days of age (Figure 10B). In addition, we calculated the disease duration in TDP-43^A315T^ mice in response to PBS or **MSN-LEP-PIO** over time (Figure 10C), and comparatively, the disease duration was longer in TDP-43^A315T^**MSN-LEP-PIO**-treated mice. Finally, motor behavior was also evaluated to control if the therapeutic use of **MSN-LEP-PIO** could change motor disease phenotype in TDP-43^A315T^ mice (Figure 10D). A two-way ANOVA revealed a significant interaction of the group by week (Figure 10D; *p* < 0.0001). This clearly indicates a modification in motor performance over time. However, TDP-43^A315T^**MSN-LEP-PIO**-treated mice showed a clear enhancement in motor performance and coordination at the end stage of the disease, indicating the potential benefit of **MSN-LEP-PIO** treatment on motor operation in TDP-43^A315T^ mice.

### 3.3 Quantification of Si Internalization

A critical point in our study is the confirmation of the therapeutic action of the nanosystems and their potential to reach different areas related to ALS disease. One of the most effective ways to confirm that MSNs were able to reach some of the key therapeutic areas is the detection and analysis of the silicon quantity in some tissues.

In this context, the accumulation of Si in selected target tissues was studied by ICP-OES. Thus, after the treatment of the animals with the silica-based nanostructured therapeutic systems, two different tissues from the treated mice (lumbar spinal cord (SC) and motor cortex (CTX)) were analyzed to determine the concentration of Si upon routinary digestion of the tissues using HNO_3_, HF, and HCl.

Silicon coming from the functionalized MSNs was detected in both CTX and SC tissues, as observed in a recent study.^36^ In all cases, concentrations above 2300 ppm were observed (Table S1), pointing to the fact that the tested materials were able to reach the target tissues and act as encapsulators or carriers of the combination of drugs leptin and pioglitazone and were able to cross the BBB.

## 4 Conclusions

A series of functionalized mesoporous silica-based systems with leptin and pioglitazone were prepared and characterized. The nanosystem was functionalized with both agents and tested as a potential therapeutic approach for the preclinical treatment of TDP-43^A315T^ ALS mice. The presence of the synthesized materials through the detection of silicon was observed in the analyzed tissues of treated animals, supporting the hypothesis that the synthesized nanosystems act as protectors of the combination of the therapeutic molecules, leptin and pioglitazone, and are able to reach key tissues associated with the potential treatment of ALS. This study, therefore, reports the first experimental data that support a therapeutic effect of the use of **MSN-LEP-PIO** in the motor function in TDP-43^A315T^ mice. However, the contribution of **MSN-LEP-PIO** in the positive motor phenotype of TDP-43-related needs additional molecular biology studies to understand the underlying mechanisms associated with the treatment with **MSN-LEP-PIO**, which may require a larger set of sizes of the samples at some other defined times and will be explored in the future by our teams. In summary, the preparation, characterization, and preliminary therapeutic study of the novel nanosystems based on the functionalization of MSNs with a cocktail of therapeutic molecules, leptin and pioglitazone, has proven the high potential of this nanoplatform for the treatment of ALS.

Current studies, already in progress in our labs, are focused on tuning the doses of the therapeutic agents by increasing the loading capacity of the silica nanoparticles for the optimization and rational design of the chemical and biological properties of the carrier to maximize their potential to cross the BBB.

## Supporting Information Available

SEM images of the material MSN, MSN-PIO, and MSN-LEP-PIO and FTIR spectra of the material series and data of the quantification of Si internalization by ICP (PDF)

## Abbreviations

CCR2: CC chemokine receptor 2
CCL2: CC chemokine ligand 2
CCR5: CC chemokine receptor 5
TLC: thin-layer chromatography
