# Supplementary material for: Design of Mesoporous Silica Nanoparticles for the Treatment of Amyotrophic Lateral Sclerosis (ALS) with a Therapeutic Cocktail Based on Leptin and Pioglitazone
Source: ACS Biomater Sci Eng. 2022 Oct 14;8(11):4838–49. doi: 10.1021/acsbiomaterials.2c00865 (PMC9667463; doi:10.1021/acsbiomaterials.2c00865)
Supplement: Supplementary file 1 — ab2c00865_si_002.pdf [file ab2c00865_si_002.pdf]

Design of mesoporous silica nanoparticles for the treatment of Amyotrophic Lateral Sclerosis (ALS) with a therapeutic cocktail based on leptin and pioglitazone

Diana Díaz-García,[a] Águeda Ferrer-Donato,[b] José M. Méndez-Arriaga,[a] Marta Cabrera Pinto,[b] Miguel Díaz-Sánchez,[a] Sanjiv Prashar,[a] Carmen M. Fernandez-Martos,[b,c] Santiago Gómez-Ruiz[a,]**

[a] COMET-NANO Group, Departamento de Biología y Geología, Física y Química Inorgánica, E.S.C.E.T., Universidad Rey Juan Carlos, Calle Tulipán s/n, E-28933 Móstoles, Madrid, Spain

[b] Neurometabolism Group, Research Unit of the National Hospital of Paraplegics (UDI-HNP), Toledo. Finca La Peraleda s/n, Toledo 45007, Spain.

[c] Wicking Dementia Research and Education Centre, College of Health and Medicine, University of Tasmania, Hobart, Tasmania, Australia.

This supporting information contains:

1) SEM images of the starting material MSN (**Figure S1**), MSN-PIO and MSN-LEP-PIO (**Figure S2**).
2) FT-IR spectra of the series (**Figure S3)**
3) Quantification of Si internalization by ICP (**Table S1**)

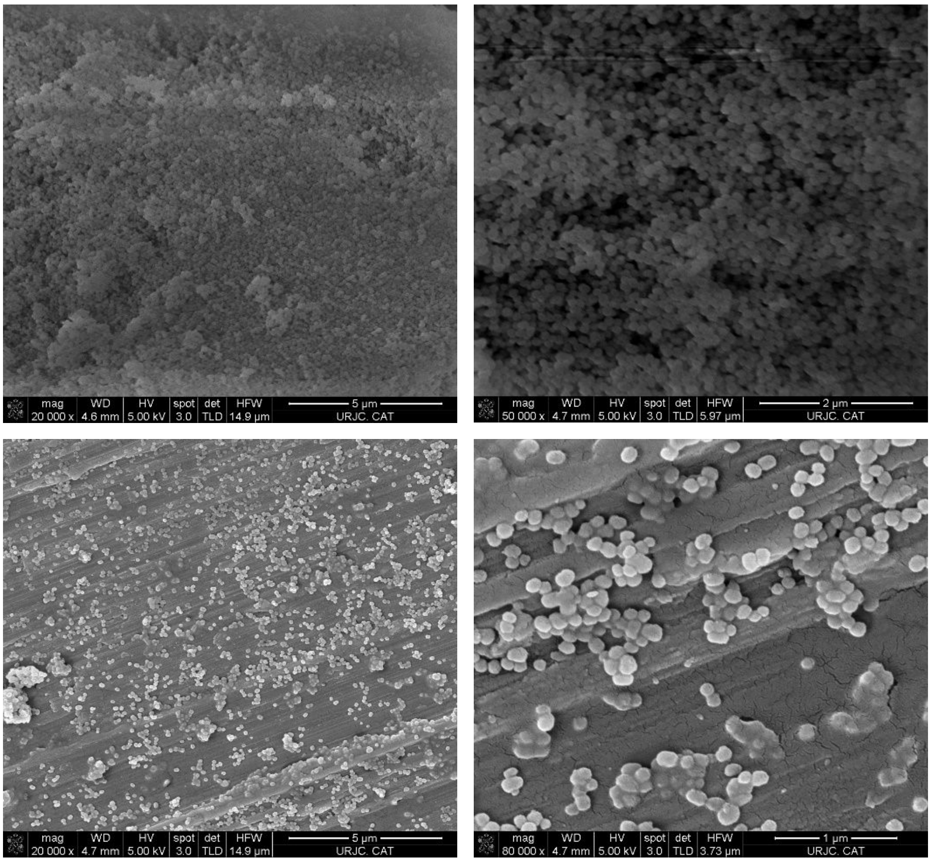

Figure S1. SEM images of **MSN** nanoparticles.

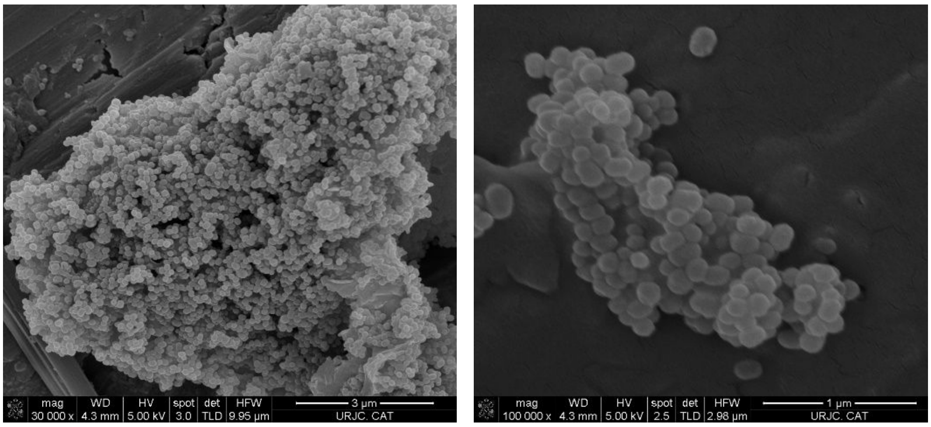

Figure S2. SEM images of **MSN-PIO** (left) and **MSN-LEP-PIO** (right).

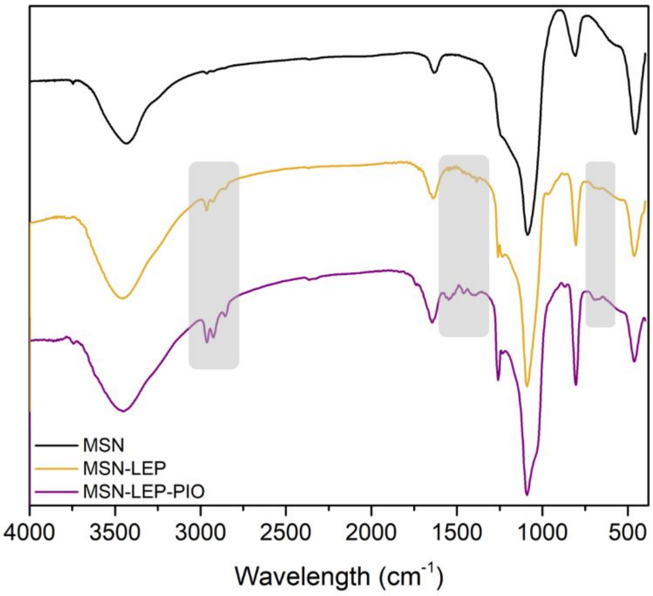

Figure S3. FT-IR spectra os MSN-LEP-PIO series.

Table S1. Si concentration accumulated in selected treated mouse tissues.

Tissue	% Si	ppm Si
TG1 FCI	35.49	7346
TG2 FCI	38.54	4817
TG3 FCI	28.72	2342
TG3 SC	72.77	3232
